# Psychological resilience and cognitive reappraisal mediate the effects of coping style on the mental health of children

**DOI:** 10.3389/fpsyg.2023.1110642

**Published:** 2023-04-03

**Authors:** Fulei Han, Ruirui Duan, Beibei Huang, Qiulin Wang

**Affiliations:** College of Physical Education, Yangzhou University, Yangzhou, Jiangsu, China

**Keywords:** coping style, psychological resilience, cognitive reappraisal, mental health, COVID-19

## Abstract

**Introduction:**

This study explored the effects of coping style and two potential intermediately factors (cognitive reappraisal and psychological resilience) on the mental health of middle school students during the normalization of epidemic prevention and control in China.

**Methods:**

Answers on questionnaires designed to assess coping style, cognitive reappraisal, psychological resilience, and mental health among 743 middle school students (386 boys, 357 girls, 241 first graders, 235 second graders, and 267 third graders) were analyzed using structural equation modeling.

**Results:**

The results showed that coping style, cognitive reappraisal, and psychological resilience directly predicted mental health. The negative effect of a negative coping style on mental health was significantly stronger than the positive effect of a positive coping style. Coping style affected mental health through the independent mediating effects of cognitive reappraisal and psychological resilience and through their chain mediation.

**Discussion:**

The use of positive coping styles by most students led to greater cognitive reappraisal, strengthened psychological resilience, and thus few mental health problems. These findings provide empirical evidence and may guide educators in the prevention and intervention of mental health problems among middle school students.

## Introduction

It has been more than 2 years since the outbreak of the COVID-19 pandemic in 2019. Although China has accumulated a lot of experience in epidemic prevention and control, the continuous variation of the SAR-CoV-2 virus brings great challenges to epidemic prevention and control. In general, the current epidemic situation in China is characterized by multiple sporadic and local outbreaks. Home isolation and centralized control continue to threaten the physical and mental health of children and adolescents. Countries or regions in which people stayed at home to slow the spread of the virus have experienced not only a decline in the academic abilities of students but also serious effects on their physical and mental health (Liu, 2020). After the COVID-19 outbreak, the incidence of psychological and behavioral problems among children and adolescents was higher than before the pandemic (Liang et al., 2020). For example, one survey found that the level of psychological problems and post-traumatic stress disorder among adolescents during the pandemic was higher than that before the pandemic. Golberstein et al. (2020) found that home learning due to the pandemic weakened the ability of schools to provide mental health services to students in a timely manner. Middle school students are in a critical period of growth and development. Their physiological functions are developing rapidly and approaching maturity, but their psychological development is not yet mature, which leads to an unbalance in their physical and mental development. In addition to the various effects of the pandemic, middle school students are also vulnerable to the impact of the external environment, which leads to various psychological problems. Therefore, it is of great practical significance to study the mental health of middle school students during the period of normalized epidemic prevention and control requirements in China to help build a school psychological service system and to improve the quality of mental health services for middle school students.

In recent years, coping has increasingly become a core issue in stress research. Coping style affects the nature and intensity of the stress response and regulates the relationship between stress and physical and mental health. Coping style refers to the coping strategies and methods adopted by individuals in the face of stressful events and is important for individuals to adapt to the environment and to form a healthy psychology. Coping styles comprise positive and negative styles (Luo et al., 2016). Cao et al. (2022) found a significant negative correlation between positive coping styles and mental health symptoms, suggesting that developing positive coping styles can effectively reduce mental health symptoms. When individuals are in risky situations, a positive coping style may weaken or cushion the negative impact on mental health, whereas a negative coping style may enhance or promote the negative impact on mental health (Stevenson and Marc, 2005; Zhang et al., 2021). A study assessing college students’ coping styles at different times during the COVID-19 pandemic found that with the continued spread of the virus, negative coping styles, such as pressure, emotion, and escape, increased, whereas positive coping styles, such as life satisfaction and task completion, showed a downward trend (Rogowska et al., 2021). Coping styles also indirectly affect mental health through mediating effects, such as sense of hope, social support, and emotional health (Wang and Miao, 2009; Mao et al., 2015; Li, 2021). Given all these data, the first hypothesis of the present study was that positive coping styles positively predict the mental health of middle school students, and negative coping styles negatively predict the mental health of middle school students.

Cognitive reappraisal is a type of cognitive change that refers to altering the understanding and personal awareness of emotional events. Cognitive reappraisal attempts to understand negative emotional events, such as anger and frustration, in a more positive way or to rationalize emotional events (Sheppes and Gross, 2011). Cognitive reappraisal is closely related to emotional experience. Individuals who often use cognitive reappraisal strategies reduce negative emotional experience and physiological reactions and have strong activation of the parasympathetic nervous system (Cheng et al., 2009). John and Gross (2004) found that people who habitually use cognitive reappraisal experience and express fewer negative emotions, more positive emotions, fewer depressive symptoms, and higher self-esteem. Cognitive reappraisers develop their own internal mental resources and improve individual life satisfaction and mental health (Krafft et al., 2019). Moreover, the use of cognitive reappraisal as an intervention strategy has been shown to alleviate the mental health problems caused by the COVID-19 pandemic and potential similar future epidemics (Zhang et al., 2021). Coping style belongs to the cognitive behavior strategy of dealing with internal and external stress, whereas cognitive reappraisal belongs to the behavioral response of dealing with internal and external stress that has strong consistency. One study found a significant positive correlation between middle school students’ positive coping styles and cognitive reappraisal. Moreover, individuals who regularly adopt positive coping styles and cognitive reappraisal are less likely to have negative stimulus bias, thereby promoting the individual’s mental health (Huang, 2020). Therefore, the second hypothesis proposed by the present study was that positive coping styles positively predict cognitive reappraisal and indirectly predict mental health through the mediating role of cognitive reappraisal. In addition, negative coping styles negatively predict cognitive reappraisal and indirectly predict mental health through the mediating role of cognitive reappraisal.

Psychological resilience refers to the ability of individuals to maintain good adaptability and positive emotions in harsh environments (Luthar et al., 2000). As a coping resource for individuals to resist external pressure, psychological resilience has been widely studied in the field of psychology (Ke et al., 2022). When individuals face various unfavorable conditions, such as adversity, frustration, and disease, the factors that help people successfully overcome difficulties is an important topic in the field of psychology. Psychological resilience includes being in adversity and making good adaptations as the two core elements (Dikec et al., 2018). Therefore, it is particularly important to study people’s psychological resilience during the normalization of pandemic prevention and control in China. Psychological resilience may reduce the perceived threat of the COVID-19 pandemic and help maintain an overall steady level of mental health (Sugawara et al., 2021). A study by McKenzie et al. (2022) found that psychological resilience is of great significance to mental health and promotes mental health. A study assessing Arab teenagers found that worry about COVID-19 had a negative predictive effect on psychological resilience and was associated with more psychological barriers (Yildirim et al., 2020). Resilience has been damaged by the COVID-19 pandemic. However, individuals with a higher level of psychological resilience are better off than those with a lower level of psychological resilience in terms of emotion regulation, life satisfaction, subjective well-being, and overall mental health (Han and Wang, 2022). A study by Yang et al. (2020) assessing senior high school students during the pandemic found that psychological resilience blocks the direct impact of psychological trauma on mental health and plays a strong role in protecting mental health. Research assessing middle students has shown a significant positive correlation between psychological resilience and coping styles. Strengthening the mental health education aimed at adolescents’ psychological resilience will help cultivate positive coping styles to thus promote overall mental health and a sense of the meaning of life (Shao et al., 2021). Another study investigating teenagers found a significant positive correlation between psychological resilience and positive coping styles: overall mental health was indirectly improved through positive coping styles, and overall mental health was indirectly decreased through negative coping styles (Campbell-Sills et al., 2006). Given all these findings, the third hypothesis proposed by the present study was that positive coping styles positively predict psychological resilience and indirectly predict mental health through the mediating role of psychological resilience. In addition, negative coping styles negatively predict psychological resilience and indirectly predict mental health through the mediating role of psychological resilience.

Psychological resilience is a complex concept that contains essential emotional factors and individual psychological processes. There is a significant correlation between cognitive reappraisal and psychological resilience (Li and Hu, 2022). As an important protective factor of psychological resilience, the frequent use of cognitive reappraisal strategies also increases psychological resilience (Zhou and Li, 2022). Mental health can be strengthened by improving cognitive reappraisal and psychological resilience (Zarotti et al., 2020). Therefore, cognitive reappraisal and psychological resilience may be used as chain mediators to promote mental health. The aforementioned research indicates that coping styles can not only directly improve mental health but also indirectly affect it by improving cognitive reappraisal or psychological resilience. Thus, the fourth hypothesis of the present study was that cognitive reappraisal and psychological resilience could mediate between positive coping style and mental health, and cognitive reappraisal and psychological resilience can also mediate between negative coping style and mental health.

**Figure 1:**
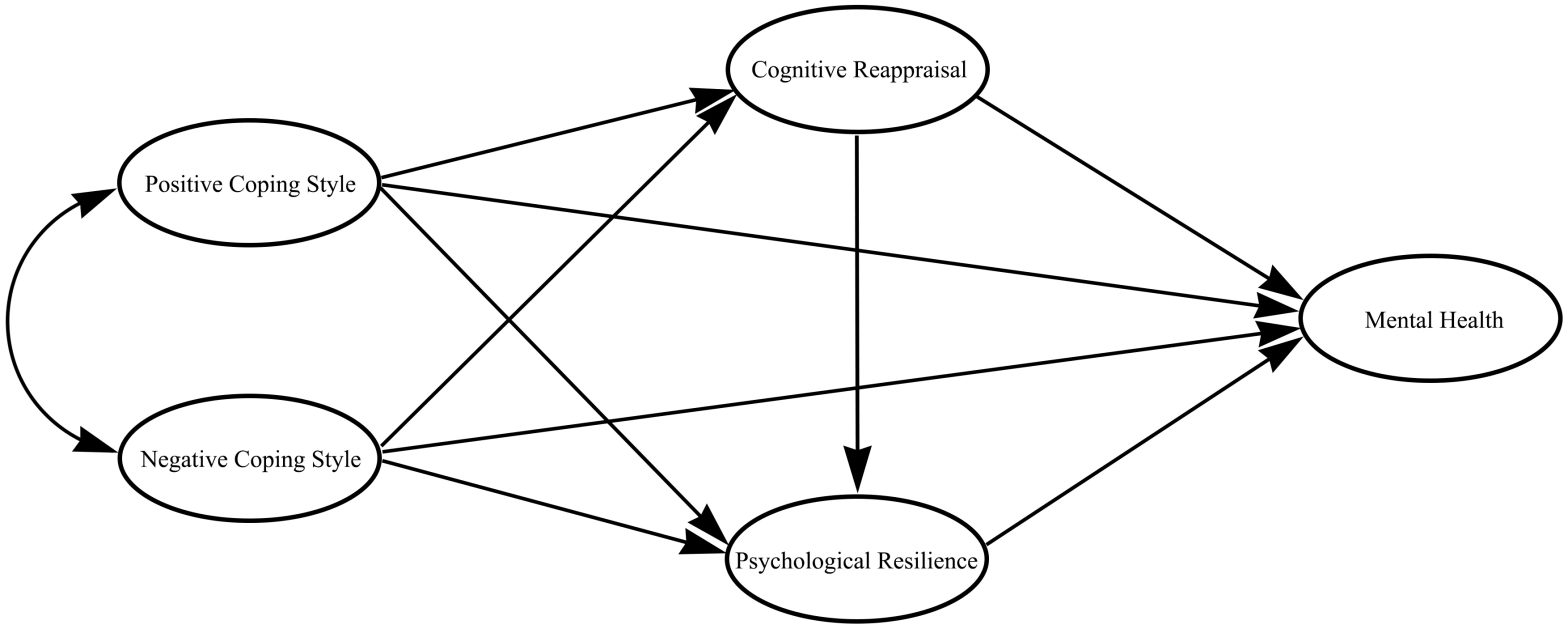
Diagram of the hypothetical model.

Although there are relationships among coping style, cognitive reappraisal, psychological resilience, and mental health, no study, to our knowledge, has reported on the mechanisms linking cognitive reappraisal and psychological resilience with positive and negative coping styles that affect mental health. Therefore, the present study explored the impact of coping styles on the mental health of middle school students during the normalization stage of COVID-19 pandemic prevention and control in China, with cognitive reappraisal and psychological resilience as chain mediators and positive coping styles and negative coping styles as independent variables. We studied the mechanisms through which coping styles impact the mental health of middle school students during this stressful time to provide suggestions and guidance for potential mental health interventions. Figure 1 shows our hypothetical model describing the intermediary mechanisms associated with the impact of coping styles on the mental health of middle school students during the normalization of COVID-19 pandemic prevention and control. Our results were largely consistent with our four study hypotheses.

## Materials and methods

### Participants and procedures

This study was approved by the Ethics Committee of Yangzhou University Medical College. From September 3 to September 15, 2022, 825 students from a middle school in Wuxi, Jiangsu Province were selected by cluster sampling. After obtaining the consent of the school leader and the head teacher, researchers distributed paper questionnaires throughout the classrooms. The participants voluntarily signed the informed consent form and then filled in the questionnaire. After the questionnaire was completed, it was immediately recovered. After removing questionnaires considered invalid due to missing data or multiple answers to a single question, 743 valid questionnaires remained, a recovery rate of 90.1%. There were 386 boys, 357 girls, 241 first graders, 235 s graders, and 267 third graders. The mean age of the included participants was 13.39 years (standard deviation = 1.10 years).

### Measures

#### Coping style scale

We used the Simplified Coping Style Questionnaire (SCSQ) compiled by Folkman and Lazarus (1988) and revised by Fang et al. (2018). The scale included two subscales: positive coping style and negative coping style. The positive coping style contained 12 questions, and the negative coping style, 8 questions. Scores ranged from 0 (“not adopted”) to 3 (“frequently adopted”). Higher scores indicated higher frequency of use for the coping style. In this study, Cronbach’s α for the positive coping style subscale was 0.868, and for the negative coping style subscale it was 0.781, indicating that both subscales had high reliability. The results of our confirmatory factor analysis showed a good model fit (*𝜒*^2^/df = 2.36, root mean square error of approximation [RMSEA] = 0.043, comparative fit index [CFI] = 0.970, Tucker-Lewis index [TLI] = 0.962, and Standardized Root Mean Square Residual [SRMR] = 0.037).

#### Cognitive reappraisal scale

We used the cognitive reappraisal scale revised by Wang et al. (2007) for adolescents from the original Emotion Regulation Questionnaire developed by Gross (2010). The scale consisted of two subscales: cognitive reappraisal and expression inhibition. The cognitive reappraisal rating scale included six items, and the scores range from 1 (“completely disagree”) to 7 (“completely agree”). Higher scores indicated a stronger emotion regulation strategy. In this study, Cronbach’s α was 0.900, indicating high reliability. The results of our confirmatory factor analysis showed a good fit model [*𝜒*^2^/df = 1.79, RMSEA = 0.033, CFI = 0.997, TLI = 0.995, SRMR = 0.012].

#### Psychological resilience scale

We used the Connor-Davidson Resilience Scale (CD-RISC) revised by Yu and Zhang (2007). The scale included three dimensions: tenacity, strength, and optimism. There were 25 questions, with scores on each question ranging from 1 (“never”) to 5 (“always like this”). Higher scores indicated better psychological resilience. In this study, Cronbach’s α was 0.953, indicating high reliability. The results of our confirmatory factor analysis showed a good fit model [*𝜒*^2^/df = 2.61, RMSEA = 0.047, CFI = 0.963, TLI = 0.957, SRMR = 0.03].

#### Mental health scale

We used the Kessler Psychological Distress Scale (K10) compiled by Kessler and Mroczek (1994) and revised by Zhou et al. (2009). The scale contained 10 questions, with scores ranging from 5 (“all the time”) to 1 (“almost none”). Higher scores indicated lower levels of mental health and more serious symptoms of mental health problems. In this study, Cronbach’s α was 0.938, indicating high reliability. The results of our confirmatory factor analysis showed a good fit model [*𝜒*^2^/df = 1.83, RMSEA = 0.033, CFI = 0.996, TLI = 0.993, SRMR = 0.014].

#### Structural equation modeling

First, confirmatory factor analysis (CFA) was conducted on each scale to obtain the composite reliability and convergent validity of each variable. The results showed that the data obtained were good. Then, correlation analysis between variables was conducted. The results showed that there was no collinearity between variables. Then, all variables were used in the hypothesis structural equation modeling for model testing. Model fit was accessed using the CFI, TLI, RMSEA, CMIN/DF (*𝜒*^2^/df), and SRMR. Model fit was acceptable when CFI > 0.90, TLI > 0.90, RMSEA<0.08, RMSEA<0.08, and 1<𝜒^2^/df < 3 (Little and Card, 2013).

### Statistical analysis

SPSS 26.0 and Mplus 8.3 statistical software were used to manage and analyze data. The results of the Harman single-factor test for common method bias (Xiong et al., 2012) conducted prior to formal data analyses showed that were nine factors with eigenvalues >1, and the variance explained by the first factor was 33.17%, which was less than the critical standard of 40%. This result indicated that there was no serious common method bias in this study.

## Results

### Reliability and validity test and descriptive statistics

As shown in Table 1, the standardized factor loadings of all variables were between 0.6 and 0.9, the composite reliability was greater than 0.7, and the convergent validity was greater than 0.4. As shown in Table 2, middle school students’ positive coping style scores and negative coping style scores were 21.99 ± 7.36 and 9.42 ± 5.17, cognitive reappraisal scores were 32.29 ± 7.56, psychological resilience scores were 83.98 ± 20.63, compared with previous studies, positive coping styles and negative coping styles did not change much, but the cognitive reappraisal and psychological resilience scores were higher (Wang and Wang, 2008; Chen et al., 2020; Han and Wang, 2022), mental health scores were 22.87 ± 8.88, according to the total score of K10, individual mental health conditions were divided into four levels: 10 ~ 19 points (grade 1, low risk of mental illness), 20 ~ 24 points (grade 2, low risk of mental illness), 25 ~ 29 points (grade 3, the risk of suffering from mental disorders is high), 30 ~ 50 points (grade 4, the risk of suffering from mental diseases is high), indicating that the mental health of middle school students as a whole is at a low-risk level (Andrews and Slade, 2001). positive coping style was negatively correlated with negative coping style (*r* = −0.125) and mental health (*r* = −0.281), and positively correlated with cognitive reappraisal (*r* = 0.503) and psychological resilience (*r* = 0.367). Negative coping style was negatively correlated with cognitive reappraisal (*r* = −0.242) and psychological resilience (*r* = −0.174), and positively correlated with mental health (*r* = 0.277). Cognitive reappraisal was positively correlated with psychological resilience (*r* = 0.599) and negatively correlated with mental health (*r* = −0.502). There was a significant negative correlation between psychological resilience and mental health (*r* = −0.373). The root mean square value of convergent validity was greater than the absolute value of the correlation between the other indicators, which, according to Bagozzi (1981), suggested that each indicator had good discrimination validity.

**Table 1:** Analysis of item reliability, composite reliability, and convergent validity.

Variable	Item reliability	Composite reliability	Convergent validity
	Standardized factor loadings		
Positive coping style	0.623–0.744	0.855	0.458
Negative coping style	0.606–0.709	0.783	0.42
Cognitive reappraisal	0.716–0.865	0.898	0.588
Psychological resilience	0.601–0.790	0.819	0.492
Mental health	0.706–0.848	0.896	0.591

**Table 2:** Descriptive statistics and correlation analysis (*N* = 743).

Variable	Mean ± SD	1	2	3	4	5
Positive Coping Style	21.99 ± 7.36	**0.677**				
Negative Coping Style	9.42 ± 5.17	−0.125^**^	**0.648**			
Cognitive Reappraisal	32.29 ± 7.56	0.503^**^	−0.242^**^	**0.767**		
Psychological Resilience	83.98 ± 20.63	0.367^**^	−0.174^**^	0.599^**^	**0.73**	
Mental Health	22.87 ± 8.88	−0.281^**^	0.277^**^	−0.502^**^	−0.373^**^	**0.769**

^**^ *p* < 0.01. Bold characters running diagonally are the root opening values of convergent validity, and the lower triangle indicates Pearson correlations of the dimensions.

### Hierarchical regression results

Based on previous research confirms that coping styles, gender, age, whether it is an only child, and place of origin are important variables affecting mental health, so these variables should be used as control variables (Table 3). With the control variables, positive coping style significantly and negatively predict mental health (*β* = −0.48, *p* = 0.018, *R*^2^ = 0.20, *F* = 12.12). Negative coping style significantly and positively predict mental health (*β* = 0.57, *p* = 0.007, *R*^2^ = 0.29, *F* = 10.19). However, the control variables included in the research did not significantly affect mental health, possibly because the samples used in this study were all from the same region. There was little difference in their living environment, education, and other factors.

**Table 3:** Hierarchical regression results.

	Model1		Model2		Model3		Model4		Model5		Model6	
Variable	E	STE	E	STE	E	STE	E	STE	E	STE	E	STE
Intercept	19.04		15.29		15.01		31.85		31.86		30.68	
Positive coping style	−0.48	−0.44	−0.34	−0.32	−0.35	−0.32	−0.32	−0.29	−0.32	0.14	−0.35	0.14
Negative coping style			0.57	0.34	0.57	0.34	0.68	0.41	0.68	0.23	0.64	0.23
Sex					0.23	0.02	0.02	0.00	−0.08	1.25	−0.06	1.23
Age							−1.40	−0.20	−1.44	0.89	−1.11	0.90
Only child									0.44	1.27	0.68	1.26
Place of origin											−1.86	1.14
R^2^	0.20		0.29		0.29		0.33		0.33		0.37	
F	12.12^*^		10.19^**^		6.67		5.80		4.58		4.39	
ΔR^2^	0.20		0.10		0.00		0.04		0.00		0.04	
ΔF	12.12		6.85		0.04		2.54		0.12		2.63	

E, coefficient; STE, standardized coefficient.

^*^p < 0.05, ^**^p < 001.

### Mediation of cognitive reappraisal and psychological resilience

Structural equation modeling was used to examine the mediating effects of cognitive reappraisal and psychological resilience on positive coping style, negative coping style, and the mental health of middle school students.

**Figure 2:**
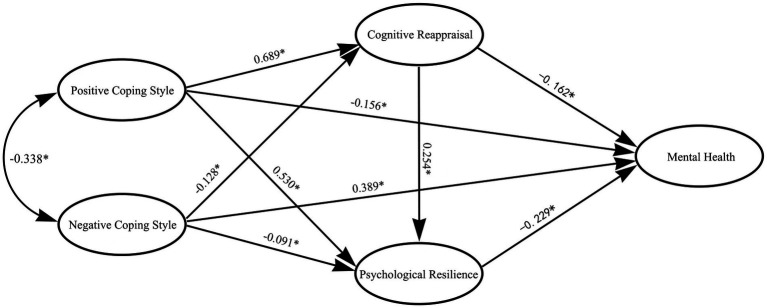
Mediation effects model.

First, the variables discussed above were tested by using a measurement model. The results showed that the fit indexes of the model were good (*𝜒*^2^/df = 2.37, RMSEA = 0.043, CFI = 0.934, TLI = 0.930, SRMR = 0.041), indicating that a full model analysis could be conducted. The structural model was then tested, and the fit indexes of the full model also indicated a good fit: *𝜒*^2^/df = 2.38, RMSEA = 0.043, CFI = 0.933, TLI = 0.929, and SRMR = 0.041. As shown in the pathway map in Figure 2: (1) positive coping styles positively predicted cognitive reappraisal (*β* = 0.689, *p* < 0.001) and psychological resilience (*β* = 0.530, *p* < 0.001) and negatively predicted mental health (*β* = −0.156, *p* = 0.028), (2) negative coping styles negatively predicted cognitive reappraisal (*β* = −0.128, *p* = 0.002) and psychological resilience (*β* = −0.091, *p* = 0.021) and positively predicted mental health (*β* = 0.389, *p* < 0.001), (3) cognitive reappraisal positively predicted psychological resilience (*β* = 0.254, *p* = 0.028), but negatively predicted mental health (*β* = −0.162, *p* = 0.028), and (4) psychological resilience negatively predicted mental health (*β* = −0.229, *p* < 0.001). These findings suggested that during the normalization of COVID-19 pandemic prevention and control, the use of positive coping styles by middle school students led to greater cognitive reappraisal and psychological resilience and fewer mental health problems.

The bias-corrected bootstrap method was used to sample 5,000 times (Table 4). The results indicated that positive coping style through cognitive reappraisal (*β* = −0.112) or through psychological resilience (*β* = −0.118) significantly affected the mental health of middle school students. In addition, positive coping style through the chain mediation effect of cognitive reappraisal and psychological resilience (*β* = −0.039) significantly affected mental health. Negative coping style through cognitive reappraisal (*β* = 0.021) or through psychological resilience (*β* = 0.021) also significantly affected mental health. In addition, negative coping style through the chain mediation effect of cognitive reappraisal and psychological resilience (*β* = 0.007) significantly affected mental health.

**Table 4:** Standardized indirect effect sizes with bias-corrected bootstrap confidence intervals.

Variable	Pathway	Effect value	Bias-corrected 95% CI		Effect size
			Lower limit	Upper limit	
Positive coping style					
	Positive coping Style → Cognitive reappraisal → Mental health	−0.112	−0.216	−0.018	12.90%
	Positive coping Style → Psychological resilience → Mental health	−0.118	−0.197	−0.060	13.60%
	Positive coping Style → Cognitive reappraisal → Psychological resilience → Mental health	−0.039	−0.081	−0.014	4.50%
	Positive coping Style → Mental health	−0.159	−0.296	−0.018	18.40%
	Total effect	−0.429	−0.506	−0.349	49.40%
Negative coping style					
	Negative coping style → Cognitive reappraisal → Mental health	0.021	0.003	0.051	2.40%
	Negative coping style → Psychological resilience → Mental health	0.021	0.005	0.047	2.40%
	Negative coping style → Cognitive reappraisal → Psychological resilience → Mental health	0.007	0.002	0.021	0.90%
	Negative coping style → Mental health	0.389	0.308	0.468	44.90%
	Total effect	0.438	0.357	0.515	50.60%
Difference					
	Direct effect difference value	0.233	0.078	0.546	
	Total effect difference value	0.009	−0.188	0.247	

The results of further analyses on the differences in the effects of the various pathways are shown in Table 4. The total difference in the effects between positive coping styles and negative coping styles was not statistically significant. However, the direct effects of positive coping style and negative coping style were significantly different. The negative impact of a negative coping style on mental health was significantly stronger than the positive impact of a positive coping style on mental health, a finding consistent with previous research (Li, 2004).

## Discussion

### Relationship between coping style and the mental health of middle school students

This study explored the relationship between coping style and the mental health of middle school students and the potential factors mediating this relationship during the normalization of COVID-19 pandemic prevention and control in China. The results showed that positive coping styles significantly and negatively predicted their mental health scores, whereas negative coping styles significantly and positively predicted their mental health scores. In other words, the higher individuals’ positive coping style level, the fewer mental health problems they experienced, but the higher the negative coping style level, the more mental health problems they experienced. The main function of coping is to adjust the impact of stressful events. Major epidemics often occur suddenly, which may lead to strong emotional responses in a short timeframe and thus may harm physical and mental health. In the process of coping, individual coping style plays a critical role and has an important impact on physical and mental health (Taylor and Stanton, 2007). An appropriate coping style can effectively relieve the psychological pressure brought by negative events, thus reducing the occurrence of mental health problems (Greenglass et al., 2006). Studies have found that during the COVID-19 pandemic, the use of negative coping styles by students, such as avoidance and acceptance, self-blame, and behavioral disengagement, was related to poor mental health, whereas their use of positive coping styles, such as positive-reframing and optimism, was related to better mental health (Liang et al., 2020). A negative coping style (problem-oriented, emotion-oriented, and dysfunctional-oriented) is an important predictor of the fear of the COVID-19 pandemic and leads to greater depression, anxiety, and stress (Kulip et al., 2022). Negative coping styles may predict mental health status over time, and mental health status may in turn predict negative coping styles over time. That is, there is a dynamic interaction between the two. Individuals who adopt negative coping styles may become fixed in an endless loop with poor mental health status. Breaking this loop may then become the only way to effectively alleviate mental health problems (Yu et al., 2019). The most effective approach to breaking the loop is to adopt a positive coping style. In the present study, the positive coping style scores of middle school students were high, and the negative coping style scores were low, indicating that the middle school students participating in this study maintained a good coping style during the normalization of COVID-19 pandemic prevention and control. However, because the use of negative coping styles may cause serious harm to mental health, families, schools, and even society as a whole should monitor the coping styles used by middle school students facing a large stressful event, such as the COVID-19 pandemic. Educators should strengthen their guidance and assist students in the use of coping styles during mental health education, improve students’ coping strategies and positive attitudes toward stressful events, and timely recognize and eliminate negative coping strategies to improve the mental health of middle school students.

### Independent mediating effects of cognitive reappraisal and psychological resilience

The present study showed that cognitive reappraisal and psychological resilience played separate mediating roles between coping style and the mental health of middle school students. The theory of cognitive reappraisal is derived from the theory of coping strategies in stressful situations. Many studies have shown that coping styles significantly affect individual cognitive reappraisal abilities during stressful events (Pascuzzo et al., 2013; Miao et al., 2021). Similar to the results of the present study, Huang (2020) found a significant correlation between coping styles, cognitive reappraisal, and negative stimulus bias among middle school students and that negative stimulus bias led to more mental health problems. Among them, the better an individual adopts cognitive reappraisal, the more likely they are to adopt positive coping styles, and the less likely they are to have negative stimulus biases, thereby promoting their mental health. Cognitive reappraisal switches the understanding of an emotion-induced situation or event to a cognitive perspective and thus changes the emotional experience. It occurs in the early stage of emotion generation, effectively changing the trajectory of the subsequent emotional response before it is completely generated. Emotion is a core indicator for evaluating individual mental health (Han and Wang, 2022). Therefore, cognitive reappraisal can predict and protect individual mental health before the occurrence of mental health problems. Thus, cognitive reappraisal plays a mediating role in the influence of coping style on the mental health of middle school students.

The results of the present study showed that psychological resilience also plays a mediating role between coping style and the mental health of middle school students. This finding provides indirect evidence for the model of the relationship between mental health and psychological resilience. The model asserts that psychological resilience is an important protective factor for mental health (Arrebola-Moreno et al., 2014). Psychological resilience enables individuals to have a strong ability to withstand pressure and to have a positive coping style and a healthy psychological level (Liu et al., 2016). Psychological resilience determines the degree of individual mental health, and mental health is the external manifestation of psychological resilience. In other words, the higher the level of psychological resilience, the better is mental health with a lower likelihood of psychological problems. Zhao et al. (2021) found that positive coping styles are significantly and positively correlated with psychological resilience, whereas negative coping styles are significantly and negatively correlated with psychological resilience. They also showed that psychological resilience affects college students’ depression levels through its impact on coping style, providing help for college students’ mental health and intervention during the COVID-19 pandemic. In the present study, middle school students had high scores for psychological resilience, an important protective factor of mental health, which may explain why the overall mental health of these students remained at low risk during the normalization of COVID-19 pandemic prevention and control (Zhou et al., 2008).

In summary, positive coping styles can protect an individual’s mental health and improve the mental health of middle school students by promoting psychological resilience, whereas negative coping styles do the opposite. Therefore, psychological resilience plays a mediating role in the effect of coping styles on the mental health of middle school students.

### Chain mediation of cognitive reappraisal and psychological resilience

The result of the present study that cognitive reappraisal significantly and positively predicts psychological resilience is consistent with previous studies (Zarotti et al., 2020). Cognitive reappraisal, an emotion regulation strategy that is initiated before the full emotion response is processed, can change the entire emotional response. The use of this strategy differs among individuals due to the differences in their abilities. Individuals who frequently use cognitive reappraisal have a high level of psychological resilience. This may be because frequent use of cognitive reappraisal may alleviate negative emotions and burnout behaviors of individuals under pressure, helping them achieve a steady state of efficacy and a healthy mindset (Chai et al., 2018) and thus promoting psychological resilience. Frequent use of cognitive reappraisal promotes more positive emotion regulation. Psychological trauma may affect mental health through the chain mediation of psychological resilience and positive emotion regulation, and psychological resilience and positive emotion regulation can block the negative impact of psychological trauma on mental health, thus protecting the mental health of high school students (Yang et al., 2020). Thus, in the present study, middle school students used more positive coping styles, which enhanced their cognitive reappraisal ability and thus improved their psychological resilience. The improvement in psychological resilience meant that the students had more protective factors to ultimately protect their mental health from the external environment. By contrast, the use of negative coping styles by middle school students not only had a negative impact on cognitive reappraisal but also led to mental health problems, such as anxiety and depression, due to a lack of protective factors. Therefore, cognitive reappraisal and psychological resilience played a chain intermediary role in the effect of coping style on the mental health of middle school students.

### Limitations and future research directions

This study had some limitations. First, this study used cross-sectional data to conduct a variety of intermediary effect analyses; thus, we could not determine whether there was a causal relationship between the variables. Therefore, in the future, longitudinal data should be used to conduct a similar effect analysis to verify the results of this study. Second, this study used only negative mental health indicators to assess the mental health of middle school students. Future studies should include positive psychological indicators, such as subjective well-being and life satisfaction, to assess the specific influences on middle school students. Third, due to the impact of the COVID-19 pandemic, the participants were students from only one geographic region. In the future, inclusion of participants from multiple regions would enable the assessment of the impact of the severity of the epidemic in each region on the mental health of students. Finally, this study assessed only middle school students. Future studies should include younger and older students to test the findings of this study.

## Conclusion

This study explored the relationship between coping styles and mental health and their mediating factors among middle school students during the normalization of COVID-19 pandemic prevention and control in China. The results showed that coping style had a significant predictive effect on the mental health of these students. Coping style affected mental health through the mediation of cognitive reappraisal and psychological resilience separately and through their chain mediation. That is, positive coping styles promoted the improvement of cognitive reappraisal abilities and thereby strengthened the level of psychological resilience to ultimately reduce the possibility of mental health problems. These findings provide empirical evidence and may guide educators in the prevention and intervention of mental health problems among middle school students.

## Data availability statement

The original contributions presented in the study are included in the article/supplementary material, further inquiries can be directed to the corresponding author.

## Ethics statement

The studies involving human participants were reviewed and approved by Ethics Committee of Yangzhou University Medical College. Written informed consent to participate in this study was provided by the participants’ legal guardian/next of kin.

## Author contributions

QW designed and conducted the research, analyzed the data, and wrote the paper. FH, RD, and BH conducted the research. FH designed the research and wrote the paper. All authors contributed to the article and approved the submitted version.

## Funding

This research was funded by 2022 Ministry of Education Humanities and Social Research Project (Grant number: 22YJE890001).

## Conflict of interest

The authors declare that the research was conducted in the absence of any commercial or financial relationships that could be construed as a potential conflict of interest.

## Publisher’s note

All claims expressed in this article are solely those of the authors and do not necessarily represent those of their affiliated organizations, or those of the publisher, the editors and the reviewers. Any product that may be evaluated in this article, or claim that may be made by its manufacturer, is not guaranteed or endorsed by the publisher.
